# The synthesis, characterization and applications of poly[*N*-isopropylacrylamide-*co*-3-allyloxy-1,2-propanediol] grafted onto modified magnetic nanoparticles

**DOI:** 10.1039/c9ra09105j

**Published:** 2020-01-22

**Authors:** Arash Alipour, Mehrnaz Babaei Shekardasht, Parvin Gharbani

**Affiliations:** Department of Chemistry, Science and Research Branch, Islamic Azad University Tehran Iran; Department of Chemistry, Ahar Branch, Islamic Azad University Ahar Iran p-gharbani@iau-ahar.ac.ir parvingharbani@yahoo.com

## Abstract

In this paper, a novel method is investigated for the extraction, determination, and delivery of ceftazidime in simulated gastric and real biological fluids such as serum plasma and urine in *in vitro* drug delivery systems. A new polymer as a nano-sorbent was synthesized by the surface grafting of [*N*-isopropylacrylamide-*co*-3-allyloxy-1,2-propanediol] onto modified magnetic nano-particles by 3-mercaptopropyltrimethoxysilane and it was characterized by FT-IR, CHN, SEM, TEM, VSM, TGA, and zeta potential analyses. The effects of main parameters such as pH, reaction time, temperature, and solvents were evaluated for ceftazidime removal. The results indicate that the newly synthesized polymer can be successfully applied for biomedical applications and drug delivery.

## Abstract 2

In this paper, a novel method is investigated for the extraction, determination, and delivery of ceftazidime in simulated gastric and real biological fluids such as serum plasma and urine in *in vitro* drug delivery systems.

## Graphical abstract

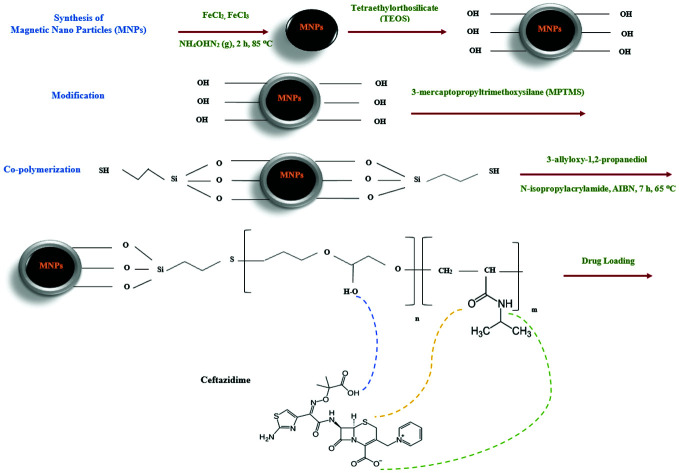

## Introduction

Ceftazidime is a semisynthetic^1^ and beta-lactam antibiotic that is widely used for the treatment of serious infections such as biliary-tract infections, bone and joint infections, cystic fibrosis, endophthalmitis, infections in immunocompromised patients, meningitis, peritonitis, pneumonia, septicemia, skin infections, and urinary tract infections caused by the Gram-positive proteus species and Gram-negative bacteria, including *Pseudomonas aeruginosa*; as a result, it is applied in infectious diseases that cause a weakened immune system.^2^ It is also used in the empirical therapy of febrile neutropenia in combination with other antibiotics.^3^ The analysis of ceftazidime is performed by HPLC,^4^ UV/Vis,^5^ GC/MS,^6–9^ LC/MS,^10,11^ IR,^12^ LC/MS/MS,^13^ capillary electrophoresis,^14–16^ and chemiluminescence^17^ in biological samples. Recently, magnetic nanoparticles (MNPs) have been widely used as drug carriers due to their size, shape, and magnetic field. Iron oxide nanoparticles are proper supermagnetic carriers. Supermagnetics are very attractive candidates in drug delivery systems due to higher biocompatibility in comparison with other systems.^18^ In these systems, a drug is easily loaded on the carrier and is also directly delivered to the target.^19,20^ As reported, iron oxide nanoparticles are not toxic for humans and cells at lower concentrations (<100 μg mL^−1^).^21^ Also, the coating of magnetic iron nanoparticles with a biocompatible polymer protects the nanoparticles from chemical reactions and hydrophobic–hydrophobic interactions in the drug delivery systems. Therefore, the aggregation is reduced and the stability is enhanced.^22^

The applications of smart biopolymers in drug delivery systems are very attractive. The main group of smart polymers is thermosensitive polymers that have two different behaviors at low and high temperatures. In fact, a part of the polymer is soluble and another part is insoluble at high temperatures.^23,24^*N*-Isopropylacrylamide (NIPAAm) is one of the well-known thermosensitive polymers that is widely used in biological applications.^25^ It possesses both hydrophilic and hydrophobic groups in its structure.^26,27^ When it enters the human body, it turns into a gel and controls the drug delivery.^28^

In this research, first, magnetic nanoparticles (MNPs) were synthesized by the co-precipitation method and modified with silane groups (MPTMS). The modified MNPs were grafted by *N*-isopropyl acrylamide as a thermosensitive polymer and 3-allyloxy-1,2-propanediol as a monomer in the radical co-polymerization method. The newly synthesized polymer with a lot of polymeric chains can load and subsequently increase the loading capacity of ceftazidime, which is used as a new nano-adsorbent in biomedical applications and drug delivery. The performance of this carrier was studied in the removal of ceftazidime from aqueous solutions and the effects of main parameters such as the contact time, pH, solvent, and temperature were evaluated. Also, it is used as a drug delivery composite in human biological fluids such as serum, plasma, and urine. The kinetics and drug delivery of the composite are investigated in simulated gastric fluids as the % release of ceftazidime.

## Result and discussion

### Characterization of MNPs@AP@NIPAAm

**Fig. 1:**
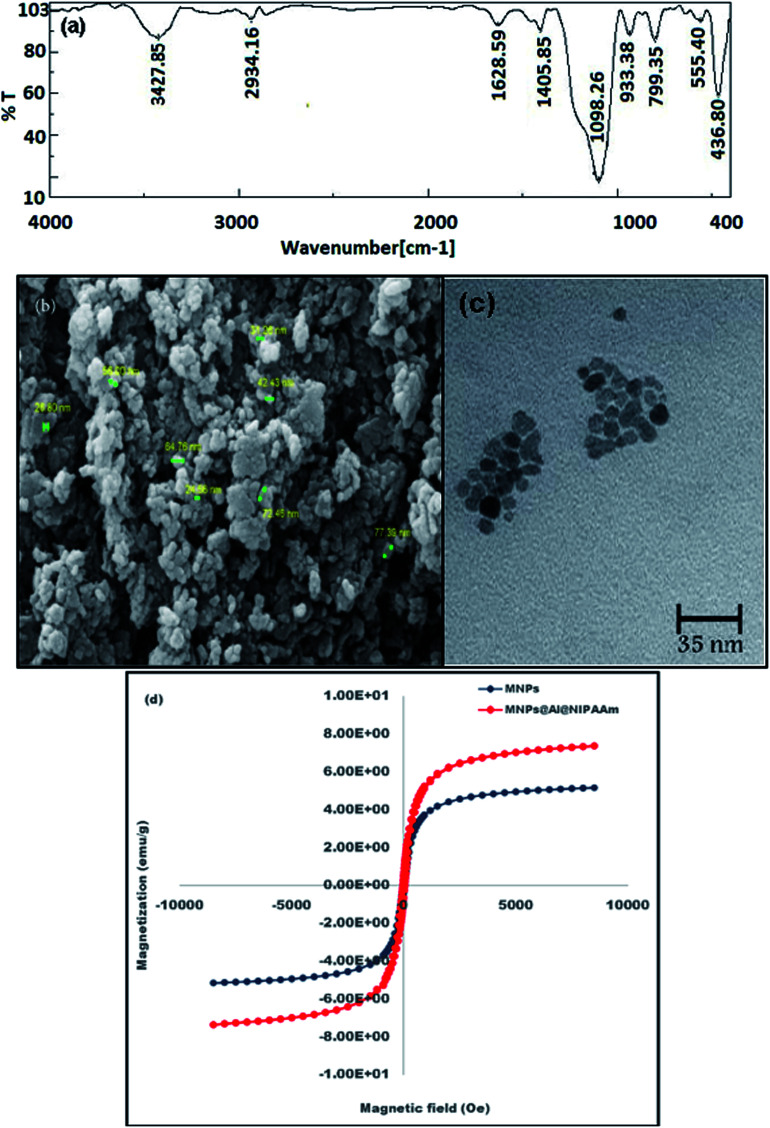
(a) FT-IR, (b) SEM, (c) TEM, and (d) VSM results of synthesized MNPs@AP@NIPAAm.

The synthesized MNPs@AP@NIPAAm was characterized by FT-IR spectroscopy. Fig. 1(a) confirms the existence of C–H asymmetric stretching (2934 cm^−1^), O–H stretching (3427 cm^−1^), and asymmetric stretching Si–O vibrations (1098 cm^−1^).^29^ The band observed at 1628 cm^−1^ is related to the plane stretching vibration of the hydroxyl group. Also, the stretching vibrations of the Fe–O groups appear at 436.80 and 555 cm^−1^. The C–H and C–O stretching vibrations are observed at 1405 cm^−1^ and 933.8 cm^−1^, respectively.^30^ The morphology of MNPs@AP@NIPAAm was determined using SEM. Fig. 1(b) shows the spherical agglomerated nanoparticles with a diameter of >100 nm. Also, it confirmed that the agglomerated surface was uniform. The TEM image of MNPs@AP@NIPAAm shows that the synthesized nano-composite is completely spherical (Fig. 1(c)). The magnetic properties of the particles were determined using a vibrating sample magnetometer (VSM). The hysteresis loops of pure and coated MNPs at room temperature are shown in Fig. 1(d). The saturation magnetization values were found to be 50.16 emu g^−1^ and 70.37 emu g^−1^ for pure MNPs and MNPs@AP@NIPAAm, respectively. This confirmed that the magnetic properties of the particles after coating rapidly increased. Therefore, they easily separated from the reaction medium in the magnetic field. The coercivity as well as the remanence value of these hysteresis loops was 0. This indicated that both samples (MNPs and MNPs@AP@NIPAAm) had superparamagnetic behavior.

**Fig. 2:**
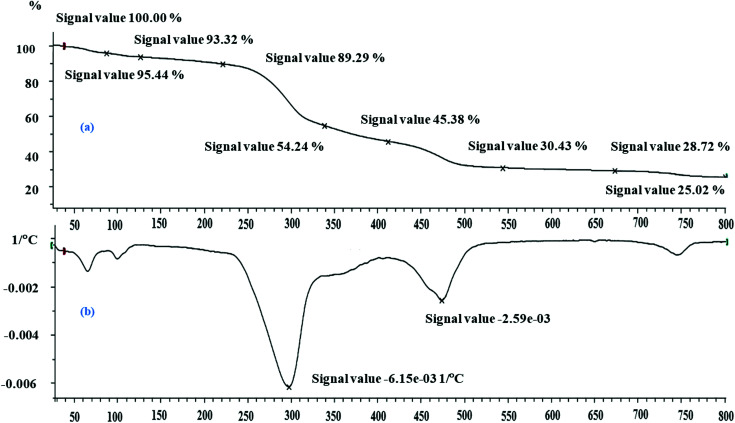
(a) TGA and (b) DTA of synthesized MNPs@AP@NIPAAm.

The thermal properties of MNPs@AP@NIPAAm were investigated by TGA-DTA (Fig. 2). The 5% weight loss up to 100 °C corresponded to the evaporation of water from the surface of the co-polymer. The 46% loss weight at 300 °C was due to the decomposition of the grafted polymeric matrix on MNPs@AP@NIPAAm. The weight loss from 300 to 550 °C was due to the decomposition of the organic part of the polymer and the desorption of the chemically immobilized polymeric matrix. As shown, there was no weight loss in the polymer from 550 °C up to 750 °C. The observed small peak in DTA (750 °C) was related to a phase change of the magnetic nanoparticles that turned into a liquid. The total weight loss was 69.57%.

To determine the surface charge of synthesized MNPs@AP@NIPAAm, its zeta potential was measured. The charge of the synthesized polymer at pH = 7.12 was zero and there was no potential for electric charge. The elemental analysis (CHN) of the synthesized co-polymer (MNPs@AP@NIPAAm) is presented in Table 1. The percentage of the C, H, and N elements increased after the polymerization of MNPs. This confirmed that the monomers were grafted successfully.

**Table 1:** Results of the CHN analysis

Elemental analysis (CHN)	C (%)	H (%)	N (%)
MNPs with silica layer (TEOS)	0.11	0.42	0.06
Modified MNPs with 3-mercaptopropyltrimethoxysilane	2.53	0.83	0.07
MNPs@AP@ NIPAA	4.39	1.12	0.71

### Adsorption and drug delivery

Effect of main parameters on the adsorption of ceftazidime onto MNPs@AP@NIPAAm.

### pH

**Fig. 3:**
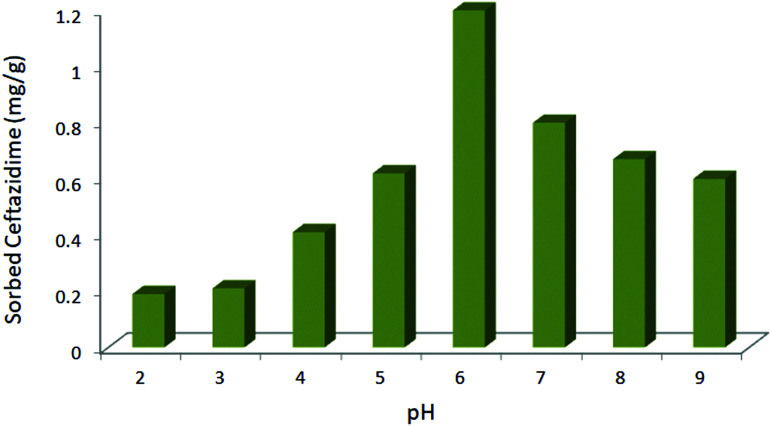
Effect of pH on the adsorption of ceftazidime. [Ceftazidime]_0_ = 100 mg L^−1^, dosage = 0.01 mg/1.5 mL, temp. 25 °C, time = 10 min.

The adsorption of ceftazidime was studied at different pH values (2–9) on a batch scale. As shown in Fig. 3, the results indicate that the maximum drug adsorption occurs at pH = 6 (optimum pH). At pH < 6, the protonation of the amino groups on ceftazidime increased. Also, the ionic form of ceftazidime and its solubility in water could be enhanced. As a result, drug extraction decreased at pH < 6. On the other hand, the magnetic nanoparticles dissolved at a higher pH (pH = 7.5).^31^ Therefore, at pH > 6, MNPs@AP@NIPAAm dissolved and the amount of the adsorbed ceftazidime decreased. As a result, the drug extraction was maximum at pH = 6.

### Solvents

The effects of different solvents on the extraction recovery of ceftazidime were investigated using methanol, methanol/acetic acid, methanol/TFA, and methanol/TFA/acetic acid to improve the elution of ceftazidime. The experiments indicated that pure methanol was the best solvent for the desorption of ceftazidime (Table 2).

**Table 2:** % Recovery of adsorbed ceftazidime on MNPs@AP@NIPAA by different solvents. [Ceftazidime]_0_ = 100 mg L^−1^, dosage = 0.01 mg/1.5 mL, temp. 25 °C, pH = 6, time = 10 min

Eluent	Recovery extraction (%)
Methanol	97.2
Methanol/acetic acid (1%)	90.3
Methanol/acetic acid (3%)	83.4
Methanol + acetic acid 5%	78.7
Methanol/TFA (1%)	59.6
Methanol/TFA (1%)/acetic acid (1%)	56.2
Methanol/TFA (1%)/acetic acid (5%)	48.3

### Contact time

**Fig. 4:**
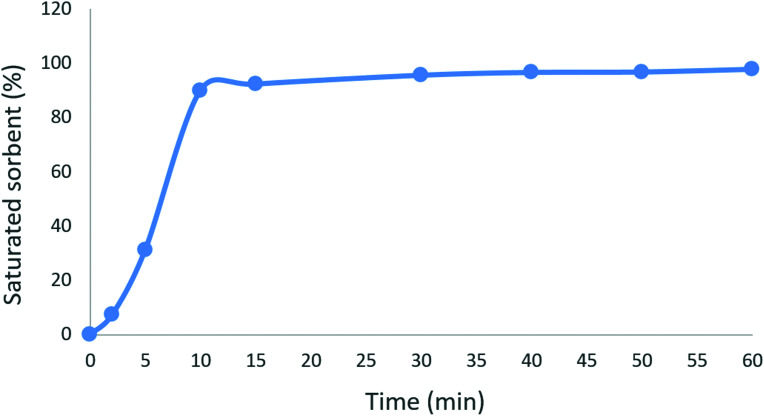
Effect of contact time on the adsorption of ceftazidime. [Ceftazidime]_0_ = 100 mg L^−1^, dosage = 0.01 mg/1.5 mL, temp. 25 °C, pH = 6.

The effect of contact time on the adsorption of ceftazidime by MNPs@AP@NIPAAm at pH = 6 is shown in Fig. 4. As shown, the adsorption of ceftazidime is fast during the initial 10 min and about 100% adsorption of ceftazidime occurs in the initial 10 min. This confirmed that the active sites on MNPs@AP@NIPAAm were accessible^25^ during the initial 10 min. Therefore, we selected 10 min as the optimum time to remove ceftazidime by MNPs@AP@NIPAAm.

### Temperature

**Fig. 5:**
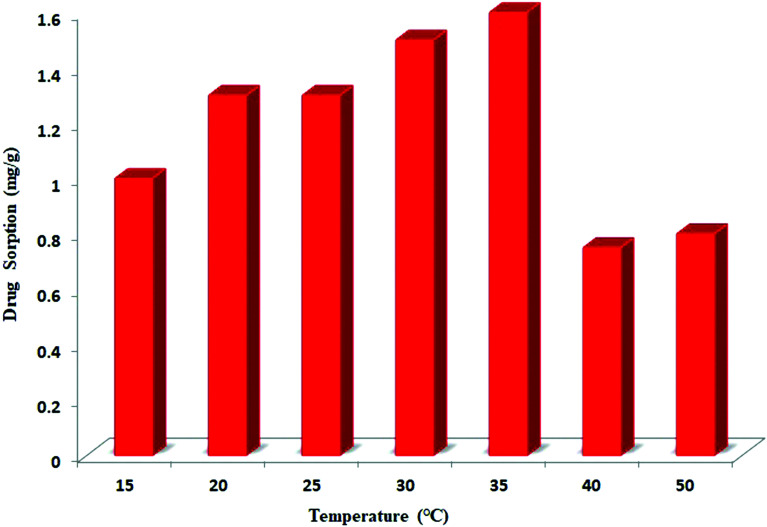
Effect of temperature on the adsorption of ceftazidime. [Ceftazidime]_0_ = 100 mg L^−1^, dosage = 0.01 mg/1.5 mL, time = 10 min, pH = 6.

The effect of temperature on the adsorption of ceftazidime onto MNPs@AP@NIPAAm was investigated from 15° to 50 °C. As presented in Fig. 5, the adsorption of ceftazidime onto MNPs@AP@NIPAAm decreases above 35 °C due to the decrease in the grafted polymer and the unavailability of the polymer to ceftazidime.

### Reusability test

**Fig. 6:**
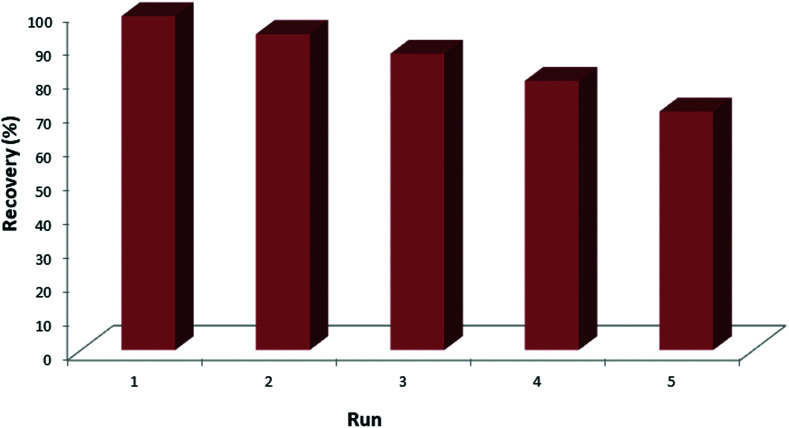
Effect of the adsorption/desorption runs on the extraction of ceftazidime. [Ceftazidime]_0_ = 100 mg L^−1^, dosage = 0.01 mg/1.5 mL, temp. 25 °C, pH = 6, time = 10 min.

The reusability of the synthesized MNPs@AP@NIPAAm was studied by conducting five adsorption/desorption runs; the results are shown in Fig. 6. The concentrations of MNPs@AP@NIPAAm and ceftazidime in each run were 6.6 mg L^−1^ and 100 mg L^−1^, respectively. After each run, MNPs@AP@NIPAAm was recovered, sonicated (20 min) and washed 3 times. As a result, after 5 adsorption runs, there was no significant reduction in the efficiency of MNPs@AP@NIPAAm. Therefore, it can be concluded that the synthesized MNPs@AP@NIPAAm is an inexpensive and effective adsorbent that can be used several times.

### Analytical validation

The limit of detection (LOD) and limit of quantification (LOQ) of ceftazidime were obtained from the calibration curve equation with 5 repetitions within a day; the values were 6.40 and 21.31 ppb, respectively.

### Applications of MNPs@AP@NIPAAm in drug delivery

In order to confirm the proposed method for the extraction and delivery of ceftazidime from various human biological fluids such as serum, plasma, and urine at optimum parameters, separation and solid-phase extraction (SPE) techniques were applied for the extraction of ceftazidime. The concentration of ceftazidime delivery was determined by HPLC. For this, human serum plasma was centrifuged for 15 min at 25 °C, filtered, and frozen at −20 °C. A certain amount of urine (50 mL) was diluted with 50 mL distilled water and spiked with ceftazidime as recommended. The recovery percentage result is indicated in Table 3 and confirms that MNPs@AP@NIPAAm has good capacity for ceftazidime extraction.

**Table 3:** Determination of ceftazidime in real biological fluid samples

Samples	Added amount spiked (μg mL^−1^)	Found (μg mL^−1^)	Recovery (%)
Plasma	1	0.92	92
Urine	1	0.17	17

### Kinetic

**Fig. 7:**
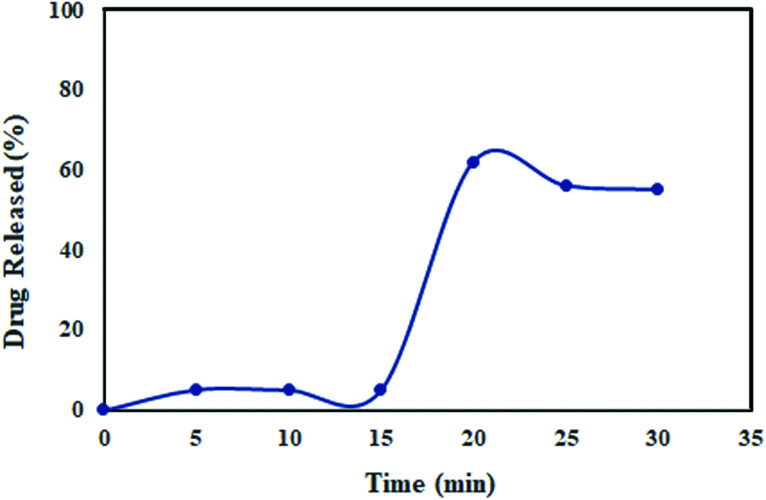
Ceftazidime release profile in the simulated gastric fluids at 37 °C and pH = 1.2.

The target drug delivery system of ceftazidime on MNPs@AP@NIPAAm was simulated in gastric fluids (pH = 1.2). To study the *in vitro* and kinetic experiments, a 0.1 N HCl buffer solution at pH = 1.2 was prepared and used as the simulated gastric fluid. A certain amount of the ceftazidime solution was loaded on MNPs@AP@NIPAAm and added in a solution of gastric fluid (pH = 1.2) with shaking at 37 °C. Every 5 min, sampling was performed until 30 min was reached; then, each sample was replaced with a fresh buffer solution and the concentration of ceftazidime was recorded. As shown in Fig. 7, the ceftazidime release in the simulated gastric fluids is about 62% with a sharp slope at 30 min. Several methods were applied to describe the release process of ceftazidime. The results of the release constants (*k*), correlation coefficients (*R*^2^), and diffusion exponents (*n*) are shown in Table 4. As a result, the release of ceftazidime was best fitted to the Korsmeyer–Peppas model; it can be concluded from the *n* value (0.356) that the mechanism of ceftazidime release is Fickian diffusion.^32^

**Table 4:** Release kinetic data of ceftazidime from MNPs@AP@NIPAA

Kinetic Model		Zero Order model^32^		First Order model^32^		Higuchi model^32^		Korsmeyer–Peppas model^32^		
Equation		*Q*_*t*_ = *k*_0_*t*		*Q*_*t*_ = 1 − e^*k*_1_*t*^		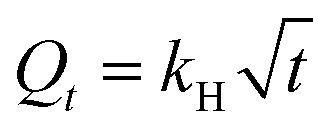		*Q*_*t*_ = 1 − *k*_KP_*t*^*n*^		
Parameters		*k*_0_	*R* ^2^	*k*_1_	*R* ^2^	*k*_H_	*R* ^2^	*k*_KP_	*n*	*R* ^2^
Value		2.78	0.884	0	0.814	16.78	0.856	16.98	0.356	0.991

## Conclusion

In this study, a new nano-adsorbent was synthesized by the radical co-polymerization process involving grafted *N*-isopropylacrylamide and it was used as a thermo-sensitive smart polymer grafted onto superparamagnetic nanoparticles. The synthesized nano-adsorbent was characterized by FT-IR, CHN, TGA, VSM, SEM, and TEM. The adsorption and target delivery of a trace amount of ceftazidime in the gastric and human biological fluids was measured by HPLC. It was proven that the chain binding between MNPs and the target polymer (NIPAAm) was appropriate for drug delivery and biomedical applications. Finally, the results of the synthesized nano-adsorbent indicate high performance and high chemical stability for the targeted and effective drug release in drug delivery systems.

## Experimental section

### Materials

3-Allyloxy-1,2-propanediol (AP) and 2,2′-azobis(2-methylpropionitrile) (AIBN) were purchased from Sigma-Aldrich. 3-Mercaptopropyltrimethoxysilane (MPTMS), *N*-isopropylacrylamide (NIPAAm), trifluoroacetic acid (TFA), tetraethylorthosilicate (TEOS), methanol, ethanol, acetic acid, H_3_PO_4_, NH_3_, FeCl_2_·4H_2_O, FeCl_3_·6H_2_O, toluene, and acetonitrile were purchased from Merck (Darmstadt, Germany). Ceftazidime was obtained from Loghman pharmaceutical company, Tehran, Iran. All reagents were of analytical grade and used without any further purification. The first stock standard solution of ceftazidime (500 mg L^−1^) was prepared in distilled water.

### Instruments

Chromatographic analysis was performed using an Agilent HPLC 1200 series equipped with a UV/Vis detector. The HPLC column was a Zorbax Extend C18 (15 cm × 4.6 mm with 3 μm particle size) from Agilent Technologies (Wilmington, DE, USA). The mobile phase consisted of 40 mL acetonitrile and 200 mL buffer at pH = 7 diluted to 2000 mL. The ultraviolet-visible (UV/Vis) spectra were recorded using a PerkinElmer/Lambda 25 UV/Vis spectrophotometer (USA). The infrared spectra were obtained using a Jasco Fourier transform infrared spectrometer (FT-IR-410, Jasco Inc., and Easton, Maryland, USA). Elemental analysis (CHN) was determined using a Thermo-Finnigan (Milan, Italy) model Flash EA elemental analyzer. Thermogravimetric analysis was carried out using TGA-50H (Shimadzu Corporation, Kyoto, Japan). The morphology of the synthesized compounds was investigated using scanning electron microscopy (SEM, EM 3200, KYKY Corporation, China) and the magnetic properties of the nanoparticles were assessed with a vibrating sample magnetometer (VSM, Homemade 2 tesla). A magnet (25 °C, 17.50 × 20 mm, 5500 Oe) was used for the collection of MNPs. The pH was recorded with a metrohm meter, model 744 (Zofingen, Switzerland). The TEM micrographs were obtained on TEM-PHILIPS CM30 TEM.

### Synthesis of functional co-polymer-grafted MNPs (MNPs@AP@NIPAAm)

Three steps were used for the synthesis of co-polymer MNPs@AP@NIPAAm: (a) the synthesis of MNPs, (b) modification of MNPs with a silane group, (c) grafted co-polymerization of 3-allyloxy-1,2-propanediol and *N*-isopropylacrylamide onto MNPs.

### Synthesis of magnetic nano-particles

The magnetic nanoparticles (MNPs) were synthesized by the co-precipitation method.^30^ Briefly, 100 mL of aqueous ammonia was gradually added to a 100 mL solution containing Fe^2+^ and Fe^3+^ (1 : 2 ratio). The solution was stirred for 2 h at 85 °C in a nitrogen atmosphere. Then, 40 mL TEOS and 80 mL ethanol/ammonia were added to the black solution. The pH was adjusted to 11 and the solution was stirred for 2 days. Finally, the obtained nanoparticles were collected, separated by a magnet, washed with ethanol and distilled water, and dried in a vacuum desiccator.

### Modification of MNPs by silane groups

MNPs (2 g) were transferred to 50 mL of a solution containing 3-mercaptopropyltrimethoxysilane (MPTMS) (5%) in toluene. The mixture was refluxed for 20 h at 90 °C. The modified MNPs were collected, separated by a magnet, washed twice with 15 mL of toluene, and dried in a vacuum desiccator.

### Graft co-polymerization of AP and NIPAAm onto modified MNPs (preparation of a new nano-sorbent)

The co-polymerization of MNPs was performed with free radical co-polymerization.^33,34^ Briefly, 2 g of modified MNPs were added to the solution containing 4 mL 3-allyloxy-1,2-propanediol, 2 g of *N*-isopropyl acrylamide as the thermoresponsive polymer, 0.2 g of AIBN as the initiator reagent, and 40 mL of ethanol. The solution was degassed, refluxed, and stirred for 7 h at 65 °C. MNPs@AP@NIPAAm was collected, separated by a magnet, washed with 40 mL ethanol, and dried in a vacuum desiccator.

### Ceftazidime adsorption/desorption

A certain amount of the ceftazidime solution (1.5 mL) was transferred into microtubes and 0.01 g of MNPs@AP@NIPAAm was added. The pH of the mixture was adjusted to 6 and the solution was shaken for 10 min. MNPs@AP@NIPAAm was collected by a magnet and the adsorbed ceftazidime was eluted with methanol. The concentration of ceftazidime was recorded by HPLC. The adsorbed amount of ceftazidime at equilibrium, *Q*_e_ (mg g^−1^), on MNPs@AP@NIPAAm was determined using eqn (1):

1 *Q*_e_ = *V*(*C*_0_ − *C*_e_)/*W*

Here, *C*_0_ and *C*_e_ (mg g^−1^) are the initial and equilibrium concentrations, respectively, *V* (L) is the volume of the solution, and *W* (g) is the mass of MNPs@AP@NIPAAm.

## Conflicts of interest

There are no conflicts to declare.

## Supplementary Material
